# Epidermolysis bullosa acquisita responsive to upadacitinib: A case report

**DOI:** 10.1016/j.jdcr.2026.09.043

**Published:** 2026-09-30

**Authors:** Tanner Harding, Yash Jani, Sara Kaye Givens, Austinn Miller

**Affiliations:** a University of Central Florida/HCA Healthcare Dermatology, Tallahassee, Florida; b Medical College of Georgia, Augusta, Georgia; c Dermatology Associates of Tallahassee, Tallahassee, Florida

**Keywords:** autoimmune blistering disease, epidermolysis bullosa acquisita, Janus kinase inhibitor, refractory dermatosis, upadacitinib

## Introduction

Epidermolysis bullosa acquisita (EBA) is a rare autoimmune blistering disorder caused by autoantibodies directed against type VII collagen, a component of anchoring fibrils at the dermal–epidermal junction. Disruption of these structures results in mechanical fragility and blister formation affecting the skin and mucous membranes. The estimated incidence of EBA ranges from 0.08 to 0.5 cases per million persons annually. Consequently, there is limited prospective data to guide management.^1^

Clinically, EBA can mimic bullous pemphigoid, linear IgA bullous dermatosis, or mucous membrane pemphigoid, leading to diagnostic delays.^2^ Histopathology demonstrates subepidermal blistering, with linear IgG and complement deposition along the basement membrane on direct immunofluorescence, and dermal-side antibody localization on salt-split skin.^1^, ^3^ There is no established gold-standard therapy for EBA. Management commonly requires prolonged systemic immunosuppression with corticosteroids and steroid-sparing agents such as mycophenolate mofetil (MMF), azathioprine, dapsone, colchicine, or cyclophosphamide. Refractory cases may necessitate intravenous immunoglobulin or rituximab. Many patients experience persistent disease activity, which may lead to corticosteroid dependence or treatment-related toxicity.^1^

Janus kinase (JAK) inhibitors have emerged as targeted immunomodulatory agents capable of suppressing multiple proinflammatory cytokine pathways implicated in autoimmune and neutrophil-mediated dermatoses. Use in autoimmune blistering diseases remains limited to small case series and reports, but data suggest potential efficacy in refractory disease.^4^, ^5^, ^6^ We report a case of severe, inflammatory EBA with a prolonged and steroid-dependent course that demonstrated marked and durable improvement following initiation of the selective JAK inhibitor, upadacitinib.

## Case report

A 68-year-old man with no significant past medical history presented with a 1-week history of progressive blistering rash, initially appearing on the feet and rapidly disseminating to involve the extremities, trunk, genitals, and oral mucosa. He denied a history of blistering or autoimmune disorders, as well as recent infections, medication changes, or constitutional or gastrointestinal symptoms.

Physical examination revealed extensive tense, painful, intensely pruritic bullae with background erythema and scattered erosions (Figs 1 and 2). Approximately 50 percent body surface area was involved at peak severity.

**Fig 1:**
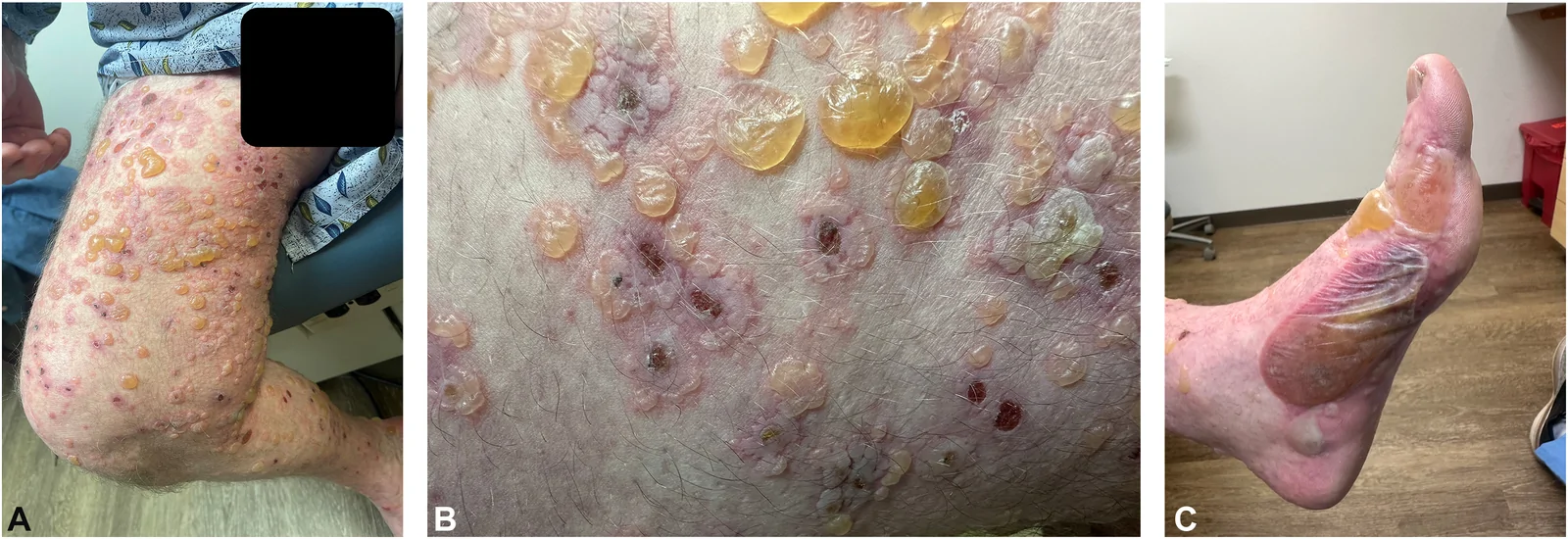
**A-C,** Generalized tense bullae with erythematous bases.

**Fig 2:**
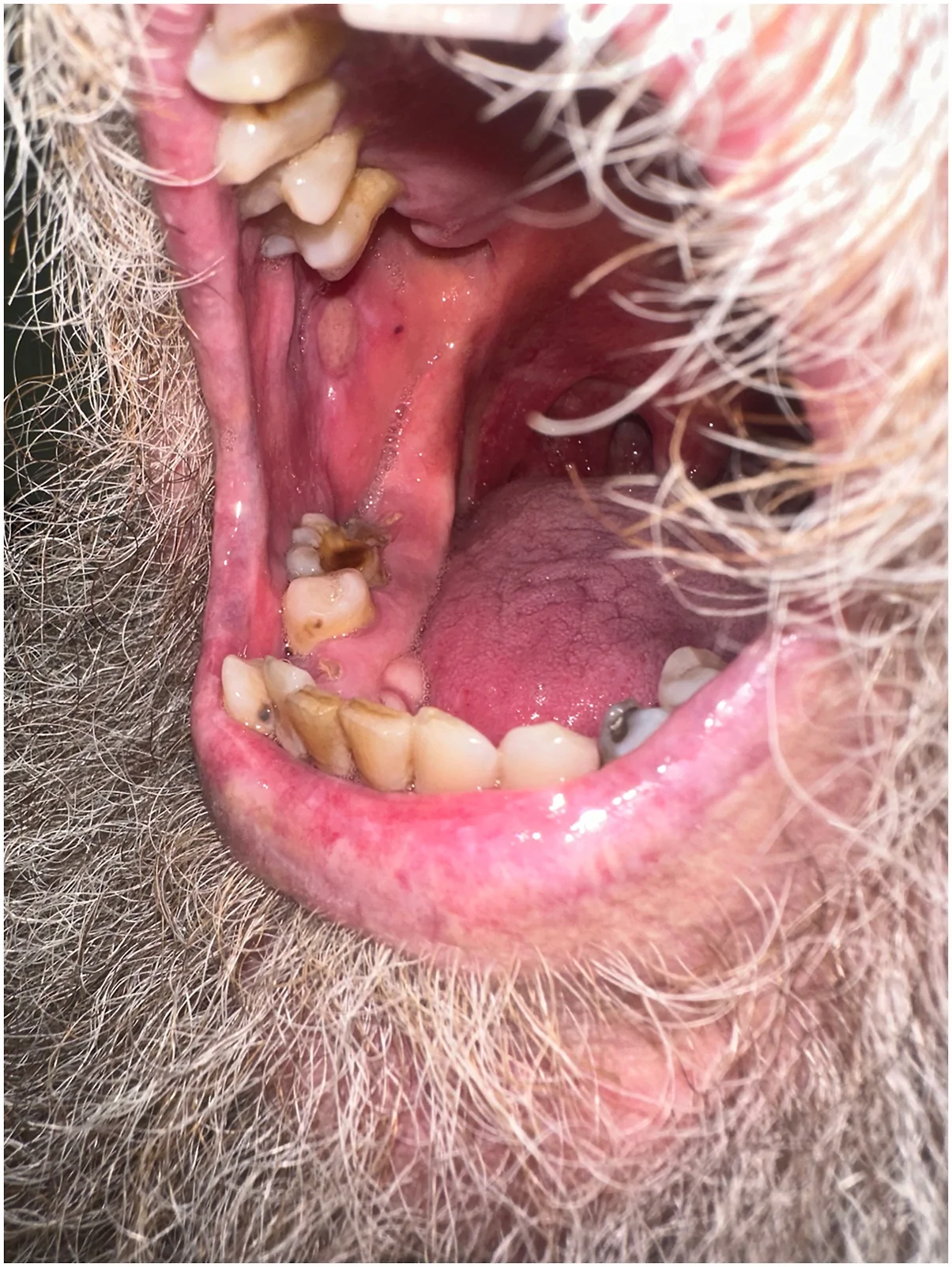
Erosion on buccal mucosa.

Punch biopsies were obtained. Hematoxylin–eosin staining demonstrated subepidermal blisters with dense inflammatory infiltrate along the dermal-epidermal junction, composed predominantly of neutrophils. Direct immunofluorescence demonstrated linear IgG and C3 deposition along the basement membrane zone. Salt-split skin testing demonstrated IgG and C3 deposition localized to the dermal side of the split, supporting a diagnosis of EBA (Fig 3). Dermal-side salt-split localization places deposits beneath the lamina densa, distinguishing EBA from bullous pemphigoid; collagen IV immunostaining was not performed, a limitation of this report.^7^

**Fig 3:**
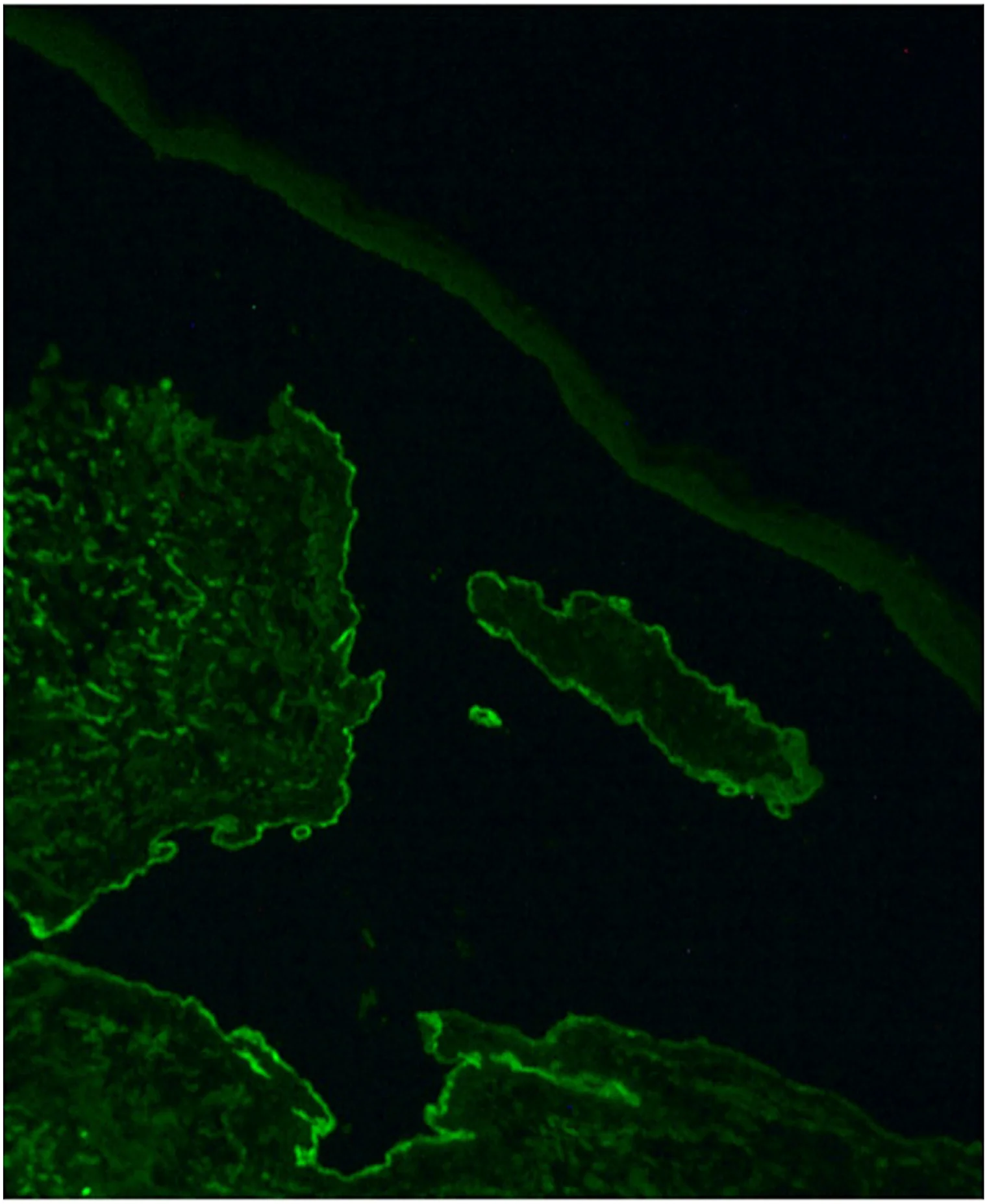
Direct immunofluorescence on salt-split skin showing IgG deposition on the dermal side of the basement membrane zone.

Serologic evaluation, including assays for BP180, BP230, desmogleins 1 and 3, and type VII collagen, was negative throughout. This does not exclude EBA: indirect immunofluorescence for anti-type VII collagen antibodies is negative in up to 40% of biopsy-confirmed cases,^7^ and this patient's prior immunosuppressive therapy, including corticosteroids and MMF, likely suppressed titers across the entire panel rather than the collagen VII assay alone. Complete blood count, comprehensive metabolic panel, antinuclear antibody, and serum and urine protein electrophoresis were unremarkable. Screening for inflammatory bowel disease and malignancy, including cross-sectional imaging, was unrevealing.

The patient was initiated on oral prednisone 60 mg daily with a planned taper, along with topical corticosteroids and comprehensive wound care. Despite initial improvement, disease burden remained substantial. MMF 1 g twice daily was initiated, along with dupilumab 300 mg every 2 weeks.

The patient experienced partial reduction in pain, pruritus, and area of involvement, but continued to develop new bullae. Disease flared with attempts to taper corticosteroids below low-dose maintenance dosing. MMF was increased to 1.5 g twice daily, and dupilimab was increased to weekly dosing. For approximately 18 months, the patient remained steroid-dependent with fluctuating but persistent blistering, despite optimizing MMF and dupilumab therapy.

At approximately 21 months, disease severity worsened dramatically. MMF was discontinued, and upadacitinib 15 mg daily was initiated while continuing dupilumab and low-dose prednisone.

Within several weeks of starting upadacitinib, the patient reported markedly reduced blistering, improved pruritus, and enhanced quality of life. Prednisone was tapered and discontinued without rebound, and dupilumab injections were spaced at increasing intervals with plans for cessation.

At 24 months, the patient demonstrated near-complete remission, with only 2 small residual bullae and without new lesions (Fig 4). He continues upadacitinib and is tapering off dupilumab without flaring. Upadacitinib, 15 mg once daily, was unchanged throughout the 24-month course; the patient had no adverse effects, including infections, cytopenias, or transaminase elevation, and oral mucosal erosions resolved alongside the skin disease.

**Fig 4:**
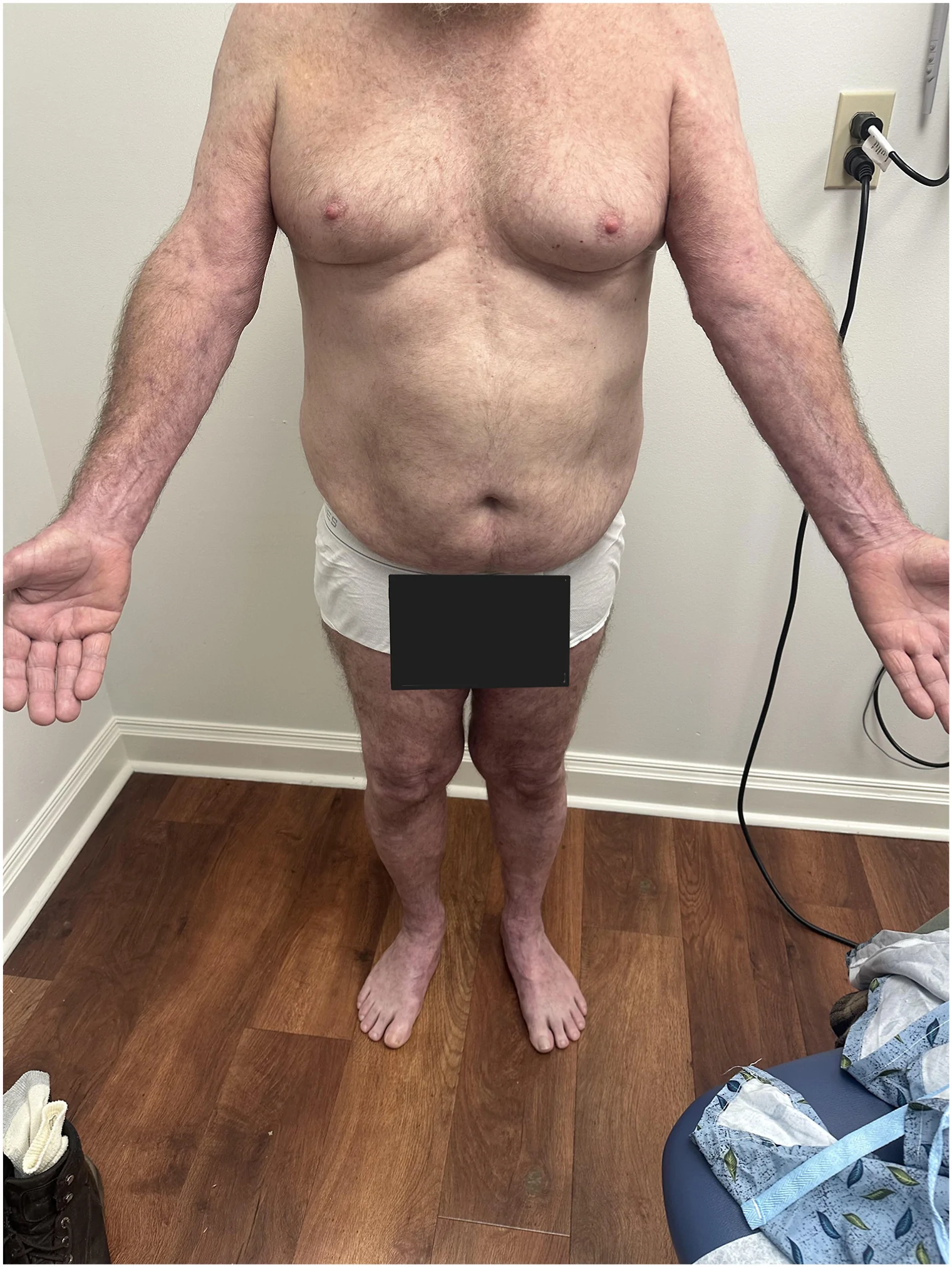
Near-complete clinical resolution of bullae and erythema following upadacitinib therapy.

## Discussion

Distinguishing EBA from other bullous entities is critical, as EBA is often more refractory and prolonged.^1^, ^3^ The eruption began acrally, on the feet, before generalizing; photographs depict this later inflammatory phase, which can mimic bullous pemphigoid, making immunopathology diagnostic here.^8^

Chronic corticosteroid use causes long-term toxicity, and steroid-sparing agents often respond incompletely. In this case, despite high-dose MMF and adjunctive dupilumab, the patient remained steroid-dependent for over a year, illustrating the limitations of conventional immunosuppression in inflammatory EBA.

By inhibiting JAK-mediated signaling, upadacitinib suppresses Th1/Th17 cytokines, neutrophil recruitment, and B-cell activation pathways implicated in inflammatory EBA.^4^ Reports have described successful use of JAK inhibitors, including tofacitinib and upadacitinib, in refractory bullous pemphigoid and EBA, either as adjunctive therapy or as a bridge to rituximab.^5^, ^6^, ^9^

Disease had recurred with every prior steroid taper over 21 months, yet remission followed upadacitinib initiation and persisted through complete corticosteroid withdrawal, a pattern inconsistent with a residual corticosteroid effect and supportive of a direct therapeutic contribution from upadacitinib.

Upadacitinib carries a boxed warning for infection, malignancy, cardiovascular events, and thrombosis1^10^; our patient had none of these, nor herpes zoster, cytopenia, transaminitis, or dyslipidemia, over 24 months. Long-term safety data for JAK inhibition in EBA remain lacking, but this case supports its potential value in this treatment-refractory disease. See Table I for treatment summary.

**Table I:** Summary of treatment course and clinical response in patient with epidermolysis bullosa acquisita

Therapy phase	Treatment regimen	Clinical response	Limitations
Initial therapy	Prednisone 60 mg daily with taper; high-potency topical corticosteroids; wound care	Partial improvement in pain and blister healing	Persistent disease activity; steroid dependence
Steroid-sparing escalation	MMFl 1 g BID → 1.5 g BID	Reduced severity and frequency of bullae	Incomplete disease control; unable to taper prednisone
Adjunctive biologic therapy	Dupilumab 300 mg every 2 wk → weekly	Improvement in pruritus and inflammation	Ongoing intermittent blistering; continued steroid dependence
Refractory disease phase	MMF + weekly dupilumab + low-dose prednisone	Disease fluctuations with recurrent flares	Progressive loss of disease control
Targeted immunomodulation	Discontinuation of MMF; initiation of upadacitinib 15 mg daily (dose unchanged thereafter)	Rapid reduction in new blister formation; marked clinical improvement	None observed
Maintenance phase	Upadacitinib monotherapy; taper and discontinuation of prednisone; spacing of dupilumab	Near-complete remission with only rare residual bullae	Long-term safety data limited; no adverse effects observed during follow-up

*BID*, Twice daily; *MMF*, mycophenolate mofetil.

## Conflicts of interest

None disclosed.
